# Leveraging functional annotations to map rare variants associated with Alzheimer’s disease with gruyere

**DOI:** 10.1101/2024.12.06.24318577

**Published:** 2024-12-08

**Authors:** Anjali Das, Chirag Lakhani, Chloé Terwagne, Jui-Shan T. Lin, Tatsuhiko Naito, Towfique Raj, David A. Knowles

**Affiliations:** 1 Computer Science, Columbia University, New York, NY, USA.; 2 New York Genome Center, New York,NY, USA.; 3 Francis Crick Institute, London, United Kingdom.; 4 Neuroscience, Icahn School of Medicine, Mount Sinai, New York, NY, USA.; 5 Systems Biology, Columbia University, New York, NY, USA.; 6 Data Science Institute, Columbia University, New York, NY, USA.

**Keywords:** Rare variants, Alzheimer’s Disease, Bayesian probabilistic model, whole-genome sequencing

## Abstract

The increasing availability of whole-genome sequencing (WGS) has begun to elucidate the contribution of rare variants (RVs), both coding and non-coding, to complex disease. Multiple RV association tests are available to study the relationship between genotype and phenotype, but most are restricted to per-gene models and do not fully leverage the availability of variant-level functional annotations. We propose Genome-wide Rare Variant EnRichment Evaluation (gruyere), a Bayesian probabilistic model that complements existing methods by learning global, trait-specific weights for functional annotations to improve variant prioritization. We apply gruyere to WGS data from the Alzheimer’s Disease (AD) Sequencing Project, consisting of 7,966 cases and 13,412 controls, to identify AD-associated genes and annotations. Growing evidence suggests that disruption of microglial regulation is a key contributor to AD risk, yet existing methods have not had sufficient power to examine rare non-coding effects that incorporate such cell-type specific information. To address this gap, we 1) use predicted enhancer and promoter regions in microglia and other potentially relevant cell types (oligodendrocytes, astrocytes, and neurons) to define per-gene non-coding RV test sets and 2) include cell-type specific variant effect predictions (VEPs) as functional annotations. gruyere identifies 15 significant genetic associations not detected by other RV methods and finds deep learning-based VEPs for splicing, transcription factor binding, and chromatin state are highly predictive of functional non-coding RVs. Our study establishes a novel and robust framework incorporating functional annotations, coding RVs, and cell-type associated non-coding RVs, to perform genome-wide association tests, uncovering AD-relevant genes and annotations.

The recent increase in available whole-genome sequencing (WGS) data has facilitated the study of rare variants (RVs), particularly in understanding their effects on complex diseases like late-onset Alzheimer’s disease (AD). AD is a neurodegenerative disorder with an estimated heritability between 59% and 74% [1]. While genome-wide association studies (GWAS) have identified over 100 loci linked to AD, with the *APOE-e4* allele as the strongest genetic risk factor, they are restricted to common variant associations [2, 3]. Despite considerable efforts to quantify the polygenic nature of AD, a significant portion of genetic heritability remains unaccounted for. Some of this missing heritability may be recovered with RVs [4]. RVs generally exhibit larger effect sizes than common variants, but their role is not yet well understood [5]. Studies have shown that integrating RVs into cumulative polygenic risk scores (PRS) can enhance predictive performance [6], but existing methods have identified fewer gene associations and have lower predictive power compared to common variant approaches. While a number of genes, including *TREM2*, *ABCA7* and *SORL1* [7], have known RV associations in AD, the majority of these findings are restricted to coding variants. As most GWAS signals lie in the non-coding genome, expanding RV association studies beyond coding variants is critical. However, the study of non-coding RVs poses challenges due to the vast number of these variants, most of which likely have no functional impact [8]. It is therefore of substantial interest to use functional annotations for variant filtering and prioritization. To develop a more robust understanding of the contributions of both coding and non-coding RVs to AD, we propose a novel method that not only weights variants according to annotations but also prioritizes functional annotations that are most trait-relevant.

Applying traditional variant-level approaches like GWAS to RVs has low statistical power due to sparsity and a high multiple testing burden due to the large number of RVs compared to common variants. To address these limitations, RV methods aggregate variants in biologically related regions, typically by gene, to increase power [9]. More recent RV methods additionally account for functional annotations to prioritize relevant variants and filter out those predicted to have no function, which otherwise reduce power [10, 11]. Despite growing efforts to accurately predict which variants will affect particular molecular phenotypes (e.g., enhancer activation, RNA splicing) [12–16], there is a limited understanding of *which functions* are the most disease-relevant. Using functional annotations that have no phenotypic associations to weight RVs can add noise to models and decrease their power. This motivated us to develop a method that learns a genome-wide mapping from functional annotations to variant importance.

Growing evidence suggests that disrupted gene regulation in central nervous system (CNS) cell types, particularly microglia, is associated with the development and progression of AD [1, 17]. The majority of RV tests are developed for coding variant associations because 1) predicting functional coding variants is comparatively straight-forward (at least for loss-of-function), 2) population-scale whole-exome sequencing predates WGS, and 3) defining non-coding regions for testing is challenging in itself. Some methods use sliding windows, but testing overlapping windows of varying sizes can result in loss of power due to multiple testing [18]. Other methods use predicted *cis*-regulatory elements (CREs), in particular enhancers and promoters, to construct testing regions [19–21]. Given their modest size (typically less than 2kb), testing individual CREs still has limited statistical power. Combining multiple CREs that regulate a gene could help address this limitation but relies on accurate predictions of enhancer-gene links. We leverage the Activity-by-Contact (ABC) model, which predicts cell-type specific enhancer-gene connectivity using chromatin state and conformation data [22]. We aggregate ABC-predicted enhancer-gene pairs to determine non-coding, cell-type and gene specific RV testing regions.

Due to the large number of genes and several million RVs found in population-scale WGS, existing methods are primarily restricted to per-gene models. This limits our understanding of disease-associated functional annotations. Most existing RV methods are explicitly, or can be viewed as, generalized linear mixed models (GLMM). We instead develop a Bayesian generalized linear model (GLM) Genome-wide Rare Variant EnRichment Evaluation, or gruyere, to model cell-type specific, non-coding RV associations on a genome-wide scale. In gruyere, a variant’s effect is a deterministic function of its annotations and the estimated AD-relevance of the gene it is linked to (if any). Our Bayesian model iteratively learns AD-relevant gene effects, covariate weights, and functional annotation importance while quantifying uncertainty, providing increased flexibility to capture the complex, hierarchical structure of genetic data. We test our model using simulation analyses and compare results to several existing RV methods. We apply gruyere to WGS data from the Alzheimer’s Disease Sequencing Project (ADSP), consisting of 7,966 cases and 13,412 controls. Our model determines splicing, transcription factor (TF) binding, and chromatin state annotations most enriched for AD-associated non-coding RVs and identifies 16 significant genes, 15 of which are uniquely identified by gruyere. Of these, four – *C9orf78*, *MAF1*, *NUP93*, and *GALNT9* – remain significant in omnibus tests.

## 2 Results

### 2.1 Genome-wide Rare Variant EnRichment Evaluation (gruyere) overview

**Fig. 1:**
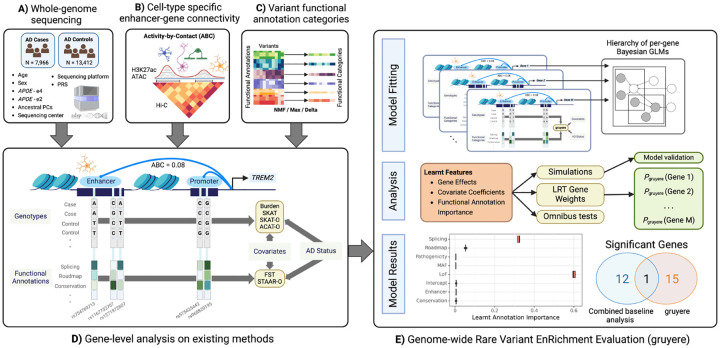
Overview of the application of gruyere to AD. Input data includes **A**) WGS and clinical information for AD cases and controls, **B**) Enhancer-gene interactions predicted by the ABC model for microglia, oligodendrocytes, astrocytes, and neurons, and **C**) variant functional annotations. **D**) Example analysis for the *TREM2* gene and microglia cell-type on existing methods. Columns represent RVs; light grey rectangles represent individual-level genotypes from WGS data for cases and controls; functional annotations for each RV are shown below genotypes; Burden, SKAT, SKAT-O, and ACAT-O are existing tests that use genotype, covariate, and AD status; FST and STAAR-O additionally use functional annotations. **E**) Workflow for gruyere. Per-gene RVs are aggregated and used for fitting the hierarchical Bayesian GLM. gruyere learns weights for covariates, genes, and functional annotations. We use simulations to assess gruyere at different heritabilities. Likelihood ratio tests are used to calculate gene-level p-values. Optionally, the gruyere p-values can be integrated with existing methods through omnibus testing.

Current RV methods rely on independent per-gene models and, therefore, cannot capture genome-wide functional annotation importance. gruyere serves as a complementary method to existing RV tests by learning trait-specific functional annotation weights, covariate coefficients, and gene effects under a Bayesian framework (Supplemental Figure 1, Table 1). Rather than modeling each gene separately, we jointly fit gruyere as a hierarchy of per-gene GLMs using stochastic variational inference (SVI) [23]. We model AD risk for each gene g,

$$\text{logit}\left(\mu_{ig}\right)=X_{i}\alpha_{g}+G_{\text{ig}}\beta_{\text{gj}}$$

where $\mu_{\text{ig}}$ is the probability of AD for individual i given the genotypes for RVs associated with gene $g,\;X_{i}$ is a vector of covariates (e.g. sex, age, *APOE-e4* genotype), and $G_{\text{ig}}$ is a genotype vector. We learn covariate weights $\alpha_{g}$ and variant effects $\beta_{\text{gj}}$. We set $\beta_{\text{gj}}$ to be a deterministic function of a learned gene effect $w_{g}$, transformed minor allele frequencies (MAFs) $w_{j}$, functional annotations Z (detailed in Methods 3.4 and Figure 1C), and learned annotation importance weights τ,

$$\beta_{\text{gj}}=w_{g}w_{j}\left(\tau_{0}+\sum_{k=1}^{q}Z_{\text{gjk}}\tau_{k}\right).$$

In our analyses, gruyere learns annotation weights τ for a range of annotations Z, including in silico mutagenesis deep learning model predictions of splicing disruption (derived as the maximum of four individual SpliceAI scores [24]) and cell-type specific TF binding and chromatin state (derived from the Enformer model [14]). A larger magnitude of $w_{g}$ indicates that disruption of gene g is associated with a higher predicted risk of AD. Similar to a burden test, gruyere assumes all variants within a gene have the same direction of effect [25, 26]. However, because our functional annotations include both loss- and gain-of-function predictions, we are able to capture additional dispersion-based signal. To ensure robust generalization of learned parameters, we split data into training (80%) and test (20%) sets, where model weights are optimized using the training set and assessed on the unseen test set. We apply gruyere to both coding and non-coding RVs for AD, defining four cell-type specific non-coding groups for AD-relevant cell types (microglia, oligodendrocytes, astrocytes, and neurons [17]) and testing each group individually.

#### Step 1. Estimating global annotation weights τ.

Fitting τ jointly across the entire genome would be 1) computationally challenging due to the large number of RVs and 2) statistically inefficient, as only AD-associated genes will contribute relevant signal. We therefore estimate τ under the gruyere model from a subset of potentially AD-relevant genes identified using a lenient significance threshold (nominal p<0.01) for the Functional Score Test (FST)[27]. We assess the robustness of gruyere estimates when selecting genes with varied significance thresholds and for a number of existing RV tests and find annotation weights τ are broadly consistent (+/− 0.02).

#### Step 2. Per-gene analysis.

Once genome-wide estimates for τ are obtained, gruyere simplifies to a logistic regression that learns covariate $\alpha_{g}$ and gene $w_{g}$ weights. We efficiently fit gruyere separately and in parallel for all genes, holding τ fixed. We perform likelihood ratio tests (LRT) to compare a covariate-only regression against combined covariate and genotype regression models to determine gene-level significance for $w_{g}$.

### 2.2 Constructing cell-type & gene specific variant sets using predicted CREs

**Fig. 2:**
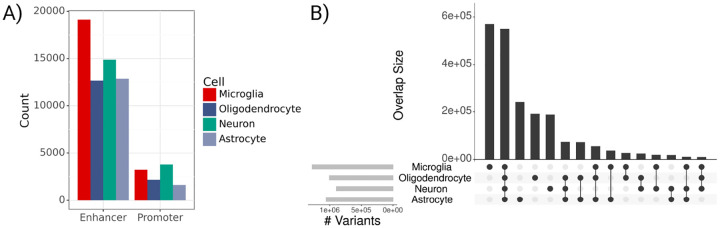
Predicted regulatory element and variant counts across cell types. **A**) Bar plot of predicted CRE counts by cell type (ABC>0.02). **B**) Upset plot of variant overlap across 4 cell types in ADSP data; Light grey bars on left indicate total RV counts for each cell type; Vertically connected dots represents groups and corresponding bars indicate variant overlap for that group.

We grouped non-coding RVs by gene and CNS cell type using the Activity-by-Contact (ABC) model (Figure 1B) [22]. The ABC model uses epigenomic profiles and chromatin conformation to determine cell-type specific enhancer-gene interactions, filtering out genes that are not expressed. We use publicly available ATAC-seq and H3K27ac ChIP-seq signals for microglia, oligodendrocytes, astrocytes, and neurons [28], as well as Hi-C averaged across ten CNS cell types to account for 3-dimensional chromatin interactions. For each gene, we analyze RVs aggregated across all CREs interacting with that gene (ABC>0.02). We test each cell type separately and also analyze rare coding variants for comparison. In total, ABC defines 70,300 CREs across all four cell types and 17,929 genes, with higher relative counts of microglia-predicted enhancers (Figure 2A). Predicted CREs frequently co-occur across cell types, with 39.4% of CREs found in more than one cell type. Promoter regions tend to have higher ABC scores than enhancers (mean ABC=0.07 vs. mean ABC=0.04), but their genomic lengths are similar, with an average length of 632bp and standard deviation of 132bp. ABC accounts for interactions of a single enhancer with multiple genes so one RV can be linked to multiple genes. In our analysis, an ABC enhancer maps to an average of 3.8 genes in microglia and between 5.4 and 5.9 genes in the other three cell types. Our non-coding variant sets contain an average of 376 RVs per gene. There are a total of 2,092,931 RVs in CREs across the four cell types, 901,570 of which are included for more than one cell type, and 550,001 that are in all four cell types (Figure 2B).

### 2.3 Simulation studies confirm accurate estimation of model parameters

**Fig. 3:**
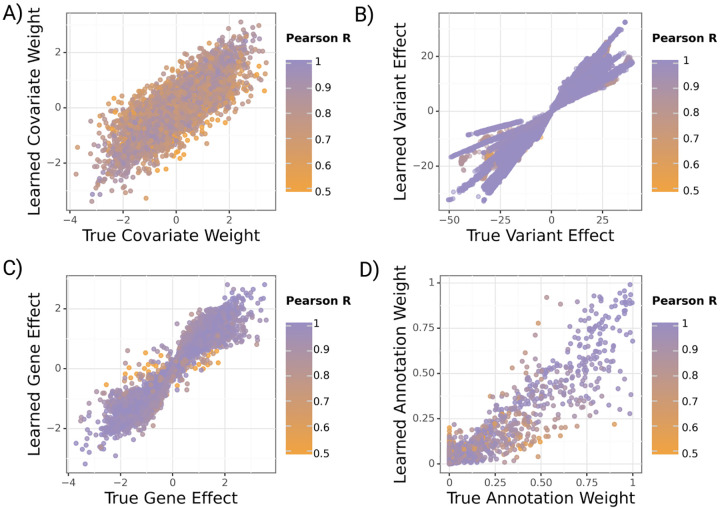
Learned versus true gruyere parameters across 100 simulations. Points are colored by the Pearson correlation coefficient of a parameter for a given simulation. **A**) Covariate regression coefficients α(c=30 covariates). **B**) Variant effect β. **C**) Gene effect $w_{g}\;(M=500$ randomly selected genes). **D**) Annotation weight τ(q=13 annotations).

We generate synthetic phenotypes (see Methods 3.5) and fit gruyere on 100 sets of simulated data with estimated heritability between 5% and 30% (detailed in Supplemental Methods [29]) using 500 randomly selected genes. We find that all variables are well recovered, with average Pearson correlations R=0.81,0.95,0.98,0.97 for $\alpha_{g},\;\beta_{\text{gj}},\;w_{g}$ and τ respectively (Figure 3). Covariates $\alpha_{g}$ have the lowest R, possibly due to correlated covariates. Average recovery across all variables remains high when varying the prior distributions (Pearson R>0.78) as well as when simulated distributions differ from the priors used during inference (Pearson R>0.66). Results are robust to the number of covariates, genes, and annotations modeled.

We analyze how simulation performance correlates with overall and genetic heritability for each simulation. This allows us to more meaningfully evaluate model performance for complex diseases like AD where estimated heritability is low. As expected, we find that gruyere is better able to recover $\beta_{\text{gj}}$ and $w_{g}$ with increased genetic heritability (Supplemental Figure 2). However, even when total heritability is as low as 5%, the minimum correlation between true and estimated parameters remains quite high (Pearson R=0.68).

### 2.4 Applying gruyere to AD WGS data reveals novel disease associations

#### Performance on ADSP WGS data.

**Fig. 4:**
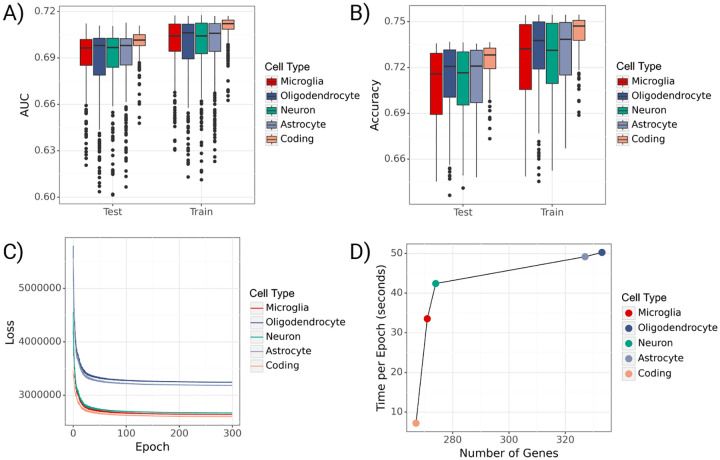
Performance of gruyere. **A**) Boxplots of per-gene AUROCs for train and test sets across cell types. **B**) Boxplots of per-gene accuracies for train and test sets across cell types. **C**) Trace ELBO loss over 300 epochs across cell types. **D**) Average training time per epoch (seconds) versus number of genes used in joint model for each cell type.

After validating model performance through simulations, we fit gruyere to the ADSP WGS data. We analyze coding and non-coding (microglia, oligodendrocyte, neuron, astrocyte) groups separately, and refer to each set as a cell type. For each cell type, we use a subset of genes for joint fitting (FST p-value < 0.01), leading to between 267 and 333 genes per cell-type. AD prediction performance is fairly consistent across non-coding variants (average test set Area Under the Receiver Operating Characteristic, or AUROC, of 0.69) and slightly higher for coding variants (average $\text{AURO}C_{\text{test}}=0.70$) (Figure 4A–B). When averaging predicted probabilities across genes, performance further improves $\left(\text{AURO}C_{\text{test}}=0.72\right)$ for all cell types. These metrics are in line with current AD literature and outperform a covariate-only regression model $\left(\text{AURO}C_{\text{test}}=0.65\right)$ [30]. There is a substantial range in prediction performance for each cell type (e.g. minimum $\text{AURO}C_{\text{test}}=0.62$, maximum $\text{AURO}C_{\text{test}}=0.71$ for microglia), highlighting the varying degrees of association with AD across genes. We find that gene-level performance is consistent across model refitting and that the loss converges reliably (4C) [31]. Fitting time increases approximately logarithmically with the total number of genes (Figure 4D). On average, it takes 37 seconds per epoch and three hours total to jointly fit gruyere across 300 epochs. Per-gene estimation is much faster, taking an average of 4.3 seconds per gene to complete.

#### Learned annotation and covariate weights.

**Fig. 5:**
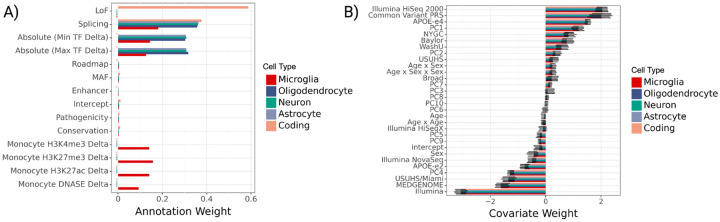
gruyere parameters learned from ADSP WGS. **A**) Bar plot of genome-wide annotation weights τ learned in jointly fit model across cell types. We denote crosses (X) to the left of bars where an annotation is not included for a cell type. **B**) Bar plot of per-gene covariate weights $\left(\alpha_{g}\right)$ learned in jointly fit model across cell types. Error bars illustrate the minimum and maximum values learned across genes.

We find that the top gruyere functional annotation weights come from splicing across all non-coding RV groups and loss-of-function (LoF) for coding variants (Figure 5A). LoF variants can be highly disruptive to gene function and are often used as a variant filtering method in gene-based tests. Therefore, it is predictable that we find gruyere places a large weight on LoF coding RVs. It is perhaps not surprising that gruyere also prioritizes RVs predicted to disrupt normal splicing, as they can substantially change the protein product or have large effects on gene dosage via nonsense mediated decay. For all non-coding regions, cell-type specific TF binding predictions from Enformer contain the next largest annotation weights. This suggests RVs associated with an increase (Max TF Delta) or decrease (Min TF Delta) in binding are predicted to have larger effects on AD, at least in AD-relevant genes. For microglia RV sets, we additionally find increased AD association for variants related to histone modification (H3K4me3, H3K27me3, H3K27ac) and DNASE in monocytes (often used as a proxy for microglia [32]). We restrict Enformer annotations to non-coding variants as they are specific to cell-types. The enhancer category has very small weights across cell-types. Since all variants included in the non-coding analyses are in putative CREs, it is perhaps not too surprising that cell-type specific enhancer annotations are lowly prioritized by gruyere.

Covariate effects are learned consistently across genes and cell types, with sequencing center, common variant polygenic risk score, and Illumina HiSeq 2000 sequencing platform as the top three covariates (Figure 5B). As expected, *APOE-e4* is learned to have a large positive risk effect while the *APOE-e2* allele has a negative (protective) effect [2]. These effects agree well with those of a simple logistic regression predicting AD status from covariates.

#### Learned gene effects and associations:

**Fig. 6:**
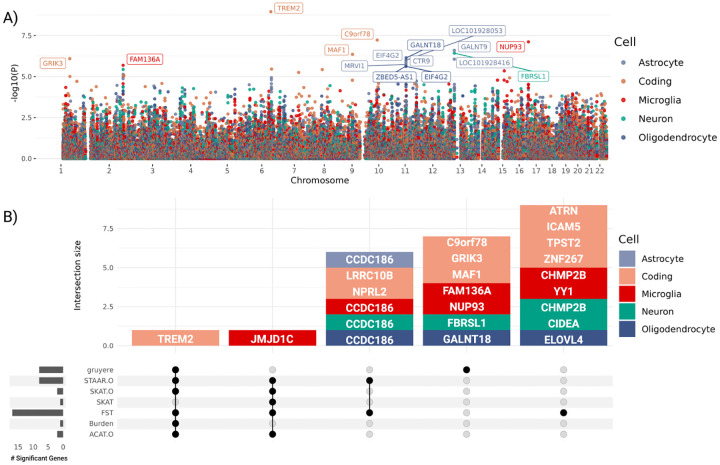
Top gruyere genes. **A**) Manhattan plot across cell types. The Y -axis shows $-{log}_{10}(p$-value) for each gene and X-axis shows gene position. Each color is a cell type. gruyere-significant genes are labeled. **B**) Stacked upset plot of significant gene overlap across all tests after pruning for coregulation. Dark grey bars on left indicate total number of significant genes for each test. Vertically connected dots represent groups and corresponding bars indicate the number of overlapping significant genes identified for that group. Each bar is colored by cell type.

Estimated gene effects $w_{g}$ are fit from a per-gene logistic regression, where we use LRTs to determine gene-level gruyere p-values (Methods 3.2). Significant genes after Bonferroni correction for each cell type are shown in Figure 6A, where a total of 16 genes reach genome-wide significance. The well-established *TREM2* RV association [33], as well as *MAF1*, *C9orf78*, and *GRIK3* are found significant for coding variants. Although not as widely recognized as *TREM2*, *MAF1* has been previously reported in association with AD [34], and *C9orf78* has been identified in an AD dementia meta-analysis [35]. *GRIK3* has emerged as a gene of interest due to the role of kainate receptors in neuroinflamation, a key feature of AD. Inflammatory responses can amplify glutamate release and disrupt receptor functioning, which may further accelerate neurodegeneration. This makes *GRIK3*, and glutamate signaling more broadly, potential targets for therapies [36, 37]. The identification of these genes by gruyere highlights their potential as candidate genes for further study in AD.

gruyere identified 12 non-coding RV associations across cell types with 2 in microglia, 6 in astrocytes, 1 in neurons, and 3 in oligodendrocytes. The most significant of these is *NUP93* (microglia), which, although not specifically linked to AD, is part of a group of nucleoporin (Nup) mutations associated with neurodegenerative disorders like AD [38]. Three significant genes, *GALNT9*, *FBRSL1*, and *LOC101928416*, are closely located on chromosome 12q24.33 and share over 80% of their ABC-predicted CREs, indicating that their associations are driven by the same set of RVs. Although variants can map to multiple genes in our model framework, making our analysis susceptible to coregulation, we are able to investigate and identify the specific CREs driving these associations. Of the overlapping promoters for these three genes, regions have higher ABC scores for *FBRSL1* (ABC = 0.20) compared to *GALNT9* (ABC = 0.04) and *LOC101928416* (ABC = 0.06), suggesting a stronger regulatory impact on *FBRSL1* and further isolating overlapping signal. FBRSL1 (neuron), has not been linked to AD, but it presents a strong candidate gene for its distinctive neuronal expression profile and involvement in neurogenesis and transcriptional regulatory networks [39]. Multiple associations (*GALNT18, CTR9, EIF&G2, ZBED5-AS1, LOC101928053*, and *MRVI1*) specific to astrocyte and oligodendrocyte cell-types are coregulated in chromosome 11p15.4, and the strongest signal, *GALNT18*, has been connected to AD in more than one study [40, 41]. After pruning coregulated signals, gruyere identifies 8 significant genes.

We identify both known and novel AD-associated risk genes with gruyere. Significant gruyere genes are associated with increased gene expression across thirteen brain tissues found in GTEx (2-sample t-test $p=9.2\times 10^{-31}$, Supplemental Figure 3A) [42]. 5 of our 16 significant genes have expression QTLs in our microglia genomic atlas (isoMiGA) that colocalize with AD or Parkinson’s disease (PD) GWAS (Supplemental Figure 3B) [43–46]. Specifically, *TREM2* and *MAF1* have significant SNPs in a recent AD GWAS [44] while *FBRSL1, EIF&G2*, and *ZBED5-AS1* are significant in a large PD GWAS [45]. AD and PD have known genetic overlap, motivating QTL colocalization of both traits [47]. Finally, we compare gruyere p-values with the Alzheimer’s Disease Variant Portal (ADVP) catalog of 956 reported AD genes, finding that gruyere yields more significant p-values for ADVP versus non-ADVP genes (2-sample t-test $p=7.1\times 10^{-9}$, Supplemental Figure 3C) [48].

##### Comparing gruyere to existing methods.

We compare pruned gruyere results with AD associations identified by a number of existing RV methods: burden test, sequence kernel association test (SKAT), optimal unified test (SKAT-O), functional score test (FST), aggregated Cauchy association test (ACAT), and variant-set test for association using annotation information (STAAR) [10, 25, 27, 49–51]. Burden, SKAT, SKAT-O, and ACAT-O tests do not include functional annotations, while FST and STAAR incorporate them (description in Supplemental Table 1 and detailed in Figure 1D for *TREM2* and microglia). We use the same set of functional annotations for FST and STAAR as for gruyere. We find that gruyere $-{\text{log}}_{10}\;$(p-values) have the highest correlation with Burden tests (Pearson R=0.86) and show moderate to high correlation with combination methods STAAR-O, SKAT-O, FST, and ACAT-O (Pearson R=0.45-0.58) (Supplemental Figure 4A). The higher observed correlation with burden tests is expected, as gruyere also assumes unidirectional variant effects within a gene. We examine overlap of significant genes across all tests and find that there is minimal overlapping signal across methods (Figure 6B). Of the 16 (8 pruned) AD associations identified by gruyere, 15 (7 pruned) are unique to gruyere, while *TREM2* (coding) is detected across all tests but SKAT where it narrowly misses significance. In total, burden, SKAT, SKAT-O, and ACAT-O tests identify only two significant associations, highlighting the importance of including functional annotations, particularly for non-coding RV associations.

#### Integrating gruyere into omnibus tests.

We combine gruyere p-values with existing methods using ACAT (Supplemental Figure 4B). Comparing *ACAT*(burden, SKAT, SKAT-O, FST, ACAT, STAAR) to *ACAT* (gruyere, burden, SKAT, SKAT-O, FST, ACAT, STAAR), we find that the inclusion of gruyere in omnibus tests boosts the number of significant associations from 12 to 16, adding *C9orf78, MAF1, NUP93* and *GALNT9*. There is no loss of power with this method, as all existing signals remain after including gruyere; we simply increase the total number of AD associations identified.

## 3 Methods

### 3.1 Data Overview

#### Whole-Genome Sequencing Data:

We analyze the latest release of WGS data from the Alzheimer’s disease sequencing project (ADSP) consisting of 21,378 unrelated individuals over the age of 65 (7,966 cases, 13,412 controls) after QC [52, 53]. We follow a standard pipeline to QC WGS data. First, we combine phenotype information across multiple cohorts and remove genetically identical duplicates (IBD $\overset{ˆ}{\pi }>0.95$) and technical replicate samples, selecting samples with the highest call rates. We prioritize phenotype information for individuals in family studies over case-control studies. Related individuals are removed using Kinship-based INference for Gwas (KING) [54], keeping AD cases where possible. In PLINK [55], we remove individuals with more than 10% genotype missingness, variants with less than 90% genotyping rate, and keep only biallelic variants with an observed MAF≤0.05. Missing genotypes are imputed as the average observed MAF. For analysis, we randomly split samples into 80% train and 20% unseen test sets, stratifying by ancestry. ADSP samples are primarily of European (N = 9,133), African (N = 5,173) and Hispanic (5,059) ancestry, with smaller South Asian (N = 1,951) and East Asian (N = 62) groups.

#### Clinical Information:

We use 30 available covariates in our model: sex, age, age^2^, age × sex, age × sex^2^, *APOE-e4* genotype, *APOE-e2* genotype, 10 ancestry principal components calculated from the 1000 Genomes Project [56], a common variant PRS [57], one-hot encoded sequencing platform (Illumina HiSeq 2000, HiSeqX, Nova Seq), one-hot encoded sequencing center (Illumina, USUHS, USUHS/Miami, NYGC, MEDGENOME, Baylor, Broad, WashU), and an intercept term (Figure 1A). Covariates are min-max scaled to a range of 0 to 1.

### 3.2 Proposed Bayesian rare variant model: gruyere

We develop gruyere, a hierarchy of per-gene GLMs (Supplemental Figure 1). We define our model jointly as

$$\text{logit}\left(\mu_{\text{ig}}\right)=X_{i}\alpha_{g}+G_{\text{ig}}\beta_{\text{gj}}$$

where $\mu_{\text{ig}}$ is the probability of AD for individual i associated with gene $g,\;X_{i}$ is a vector of covariates, and $G_{\text{ig}}$ is a vector of genotype dosages for each RV. Covariate coefficients $\alpha_{g}$ are modeled from prior,

$$\alpha_{g}\sim \text{Normal}\left(0,1\right).$$

The key innovation in the gruyere model is the construction of per-variant genetic effects for gene g, or $\beta_{\text{gj}}={\left(\beta_{\text{gj}1},\ldots ,\beta_{\text{gjp}}\right)}^{T}$, which is defined as the product of gene effects, transformed MAF, and weighted functional annotations. Of note, if a variant j is included in the RV set for both genes $g_{1}$ and $g_{2}$, the variant effect can differ for $\beta_{g_{1}j}$ and $\beta_{g_{2}j}$. We define $\beta_{\text{gj}}$ as

$$\beta_{\text{gj}}=w_{g}w_{j}\left(\tau_{0}+\sum_{k=1}^{q}Z_{\text{gjk}}\tau_{k}\right)$$

where $w_{g}$ are gene importance weights, $w_{j}$ are variant weights based on observed MAF as suggested by Wu et al.[49], τ are genome-wide annotation importance scores, and $Z_{gjk}$ is a scaled functional annotation k for RV j and gene g. The variables are modeled as,

$$w_{j}=\text{Beta}\left(\text{MA}F_{j}|1,25\right)\;w_{g}\sim \text{Normal}(0,1)\;\tau \sim \text{Dirichlet}\left(1_{q}\right)$$

For each gene, we use a Bernoulli likelihood to sample $\sigma \left(X_{i}\alpha_{g}+G_{\text{ig}}\beta_{\text{gj}}\right)$, and aggregate loss across each g∈M. Learned parameters are $\alpha_{g},\;w_{g}$, and τ. We select a Dirichlet prior for annotation weights τ to ensure identifiability between τ and $w_{g}$. Without constraining τ to a fixed sum, $w_{g}$ can be swapped for $w_{g}/c$ and τ for cτ for any positive constant c without changing the likelihood, leading to non-identifiability between gene and annotation weights.

We approximate the true posterior distribution for gruyere by minimizing the Kullback-Leibler (KL) divergence, which is equivalent to maximizing the Evidence Lower Bound (ELBO) [31]. To maximize the ELBO, we use SVI, implemented in the pyro probabilistic programming language [58]. We approximate the posterior distribution of latent variables $\alpha_{g}$ and $w_{g}$ with mean field normal distributions (AutoNormal guide), while optimizing annotation weights τ as point estimates with a Delta distribution (AutoDelta guide). We apply the Adam optimizer, a learning rate of 0.1, train for 300 epochs, and draw 50 samples from the posterior to estimate standard deviations of the learned parameters. We explore different prior distributions for all key parameters.

Once global τ is learned, we streamline gruyere with a per-gene analysis. Holding τ fixed, our model simplifies to a logistic regression where only $\alpha_{g}$ and $w_{g}$ are estimated. gruyere efficiently computes gene-level p-values using LRTs comparing a covariate-only regression to a combined covariate and genotype regression model:

$$LR_{g}=-2\times \left(LL_{{\text{combined}}_{g}}-LL_{\text{covariate}\;\text{only}}\right),\;\text{df}=1$$

$$\text{gruyere}\;p\text{-value}(g)=P\left(\chi^{2}>LR_{g}\right)$$

where LL are the log-likelihoods for each logistic regression. For each cell type, we use Bonferroni correction to define the p-value significance threshold:

$$p<\frac{0.05}{\#\;\text{genes}\;\text{per}\;\text{cell}\;\text{type}}\Rightarrow 2.88\times 10^{-6}<p<3.64\times 10^{-6}$$

### 3.3 Cell-type specific RV gene set prediction using the ABC model

We calculate enhancer-gene connectivity using publicly available ATAC-seq and H3K27ac ChIP-seq data for human microglia, oligodendrocytes, astrocytes, and neurons [28]. We apply ABC to this data following the guidelines and default parameters provided at https://github.com/broadinstitute/ABC-Enhancer-Gene-Prediction. This involves first calling candidate peak regions for the ATAC-seq DNase hypersensitive sites (DHS) using MACS2 (peakExtendFromSummit = 250, nStrongestPeaks = 150000). Then we quantify enhancer activity as the geometric mean of the read counts of DHS and H3K27ac ChIP-seq in candidate enhancer regions. Lastly, we compute the ABC score using averaged Hi-C data (hic_resolution = 5000) fit to the power-law model. The omics data is aligned to hg19, so we converted the ABC-predicted start and end positions of enhancers to hg38 for analysis. For each gene and separately each cell type, we aggregate all elements E for gene G that have an ABC≥0.02 and extract RVs from within these regions to determine our cell-type specific non-coding RV gene sets.

### 3.4 Calculating functional annotation groups

We use a range of variant-level functional annotations primarily from the Whole Genome Sequencing Annotation database [59]. Annotations with greater than 5% missingness in our RVs are removed, resulting in 50 coding variant and 52 non-coding variant functional annotations listed in Supplemental Table 2. To reduce dimensionality of related annotations while accounting for their diverse biological effects, we apply non-negative matrix factorization (NMF) to summarize groups of related annotations, inspired by STAAR [10]. We use NMF to retain interpretable directional scaling of annotations. Based on correlation structure and a priori knowledge, we define six major functional categories – splicing, conservation, integrative deleterious predictions, brain-related Roadmap epigenetics, population-specific MAF, and enhancer activity [15, 60, 61]. Because the splicing annotation group is derived from four SpliceAI predictions that are not highly correlated and sparsely distributed, we instead use the maximum score for this category. We also include a binary LoF prediction calculated with Loss-Of-Function Transcript Effect Estimator (LOFTEE) [62] for coding variants along with an intercept term. All annotations are scaled between 0 and 1, where a larger value represents increased predicted variant function.

#### Deep Learning Delta Scores:

For all four cell types, we include additional cell-type specific functional annotations: absolute maximum and absolute minimum TF delta scores derived from Enformer [14], a deep learning genomics model. We calculate variant delta scores for 5,318 functional genomics assays. The Enformer model predicts read counts (in 128 BP bins) of these assays as a function of 196,608 BP input DNA sequence. For a particular variant, we compare the model output of the reference sequence, centered around the variant position, with the output of the alternative sequence which replaces the reference allele with the alternative allele. For a particular genomics assay, the delta score is the difference between the sum of reference sequence predictions for the middle 32 bins and the sum of the alternate sequence predictions for the same bins. We normalize these scores by first calculating the delta scores for the approximately 18 million variants from the UK Biobank cohort used in PolyFun [63, 64], and then Z-score normalize each assay according based on this collection of variants. We apply this normalization to the delta scores used in our analysis. We aggregate delta scores to determine composite maximum and minimum predictions for each variant, highlighting the delta scores of only the enriched TFs within each of the four cell types ([28]). For microglia non-coding variant sets, we additionally use delta scores for 4 epigenetic marks (H3K4me3, H3K27ac, H3K27me3, and DNASE) for monocytes, a proxy for microglia.

### 3.5 Data Simulation

We use simulations to evaluate gruyere performance. We randomly sample values for each parameter and use these simulated variables in the GLM framework, $\text{logit}\left(\mu_{\text{ig}}\right)=X_{i}\alpha_{g}+G_{\text{ig}}\beta_{g}$. The real ADSP genotypes $G_{\text{ig}}$, covariates $X_{i}$, and functional annotations $Z_{gjk}$ along with simulated parameters $\alpha_{g}^{S},\;w_{g}^{S}$, and $\tau^{S}$, generate simulated phenotypes $Y_{\text{ig}}^{S}$. Simulations are restricted to a maximum estimated heritability of 30% to realistically evaluate complex diseases. For each simulation, we draw gruyere parameters from the following distributions:

$$\alpha_{g}^{S}\sim \text{Normal}(0,1),\;w_{g}^{S}\sim \text{Normal}(0,1),\;\tau^{S}\sim \text{Dirichlet}\left(1_{q}\right)$$

We define $\beta_{\text{gj}}$ in the same way, simply using simulated variables:

$$\beta_{\text{gj}}^{S}=w_{g}^{S}w_{j}\sum_{k=1}^{q}Z_{gjk}\tau_{k}^{S}$$

Using this combination of true data and simulated parameters, we sample synthetic phenotypes $y_{g}^{S}$ from a Bernoulli distribution. We perform 100 simulations on M=500 randomly selected genes. In general, we sample gruyere parameters from the same distribution that they are learned. We have also tested model performance when simulated data comes from a different distribution than its learned counterpart (e.g. sampling $w_{g}$ from a Normal distribution in simulations but fitting from a Gamma prior).

## 4 Discussion

We develop gruyere, a functionally-informed RV association test that fits a hierarchy of Bayesian GLMs to estimate genome-wide functional annotation importance, gene effects, and covariate coefficients. gruyere builds upon existing RV methods with two key advancements: 1) a genome-wide approach that enables trait-specific weighting of functional annotations, and 2) a flexible, powerful and calibrated probabilistic framework that estimates uncertainty. We incorporate an innovative methodology for analyzing RVs in the non-coding genome. Using the Activity-by-Contact model, we predict cell-type-specific enhancer-gene connectivity from chromatin state and conformation data, aggregating predictions by gene to define interpretable non-coding RV testing regions. We use in silico mutagenesis under state-of-the-art deep learning models of pre (SpliceAI) and post (Enformer) transcription gene regulation to predict RV effects. Simulation analyses validate gruyere and show it is able to recover ground truth parameters across diverse model specifications and even for realistically low heritability.

We apply gruyere, along with a number of established RV association tools, to the most recent WGS release from ADSP. Our analysis identifies both known (e.g., *TREM2*) and novel (e.g., *NUP93*) candidate AD genes. Specifically, gruyere uniquely identifies 15 genes, of which *C9orf78, MAF1, NUP93* and *GALNT9* remain significant in aggregated Cauchy tests. Our analysis additionally provides an improved understanding of AD-relevant functional annotations. gruyerez confirms the expectation that LoF is the most informative annotation for coding variants, but additionally finds deep learning-based predictions for splicing, TF binding and chromatin state are highly predictive of functional non-coding RVs.

We use ancestry principal components as covariates to account for population diversity, but one area for future work would be integrating a random effect term to better account for relatedness and population structure [65]. Another possible extension to gruyere would be incorporating gene-level features as priors [66]. While we focus our analysis on AD, gruyere can be applied to any complex disease with sufficient WGS data. As the quality of functional annotations continues to improve, gruyere will become an increasingly valuable tool for identifying disease-associated genes and annotations.

## Supplementary Material

Supplement 1

Supplement 2

## Figures and Tables

**Table 1:** Summary of gruyere Variables

Variable	Shape	Description
Y	n×1	Phenotypes for n samples
X	n×c	c Covariates for n samples
$G_{g}$	$n\times p_{g}$	Genotypes for p variants in gene g for n samples
$Z_{g}$	$p_{g}\times q$	q Functional annotations for p variants in gene g
$\alpha_{g}$	c×1	c Covariate coefficients
$\beta_{\text{gj}}$	$p_{g}\times 1$	Variant effect sizes for p variants in gene g
$w_{j}$	$p_{g}\times 1$	Variant weight Beta(${\text{MAF}}_{j}$∣1, 25)
$w_{g}$	1×1	Gene importance weights
τ	q×1	Functional annotation weight

## Data Availability

This paper uses the ADSP Release 4 WGS data and AD phenotype data.
